# Conditional Deletion of All Neurexins Defines Diversity of Essential Synaptic Organizer Functions for Neurexins

**DOI:** 10.64898/2026.07.15.738748

**Published:** 2026-07-17

**Authors:** Man Jiang, Yang Li, Margarita Artyukhova, Li Zhang, Shu Luo, Weiran Zhan, Bo Zhang, Ozgun Gokce, Thomas C. Südhof

**Affiliations:** a Department of Molecular and Cellular Physiology, Stanford University Medical School, 265 Campus Drive, Stanford, CA 94305, USA; b Howard Hughes Medical Institute, Stanford University Medical School, 265 Campus Drive, Stanford, CA 94305, USA; c Department of Physiology, School of Basic Medicine and Tongji Medical College, Huazhong University of Science and Technology, Wuhan 430030, China

**Keywords:** Neurexin, Parvalbumin, Somatostatin, Inhibitory synapse, Calcium transients, Biological Sciences/ Neuroscience

## Abstract

Presynaptic neurexins are key regulators of synapse properties that arguably represent the best-studied synaptic adhesion molecules. Despite thousands of papers, however, no direct comparison of overall neurexin functions in different types of synapses is available. A decade ago, we provided such an analysis but we recently retracted this paper because four images contained microduplications that, although without discernible impact on the paper’s conclusions, could not be corrected. As a result, the scientific community lost access to primary data that established the fundamental principle that neurexins perform profound but distinct functions in different types of synapses. In the present study, we have therefore reanalyzed the original raw data and expanded their conclusions with new experiments to document in a single study the basic contributions of neurexins to different synapses. Using triple conditional knockout mice that target all neurexins except for Neurexin-1γ and applying neuron-specific manipulations combined with slice electrophysiology, two-photon Ca^2+^ imaging and immunohistochemistry, we focussed on excitatory climbing-fiber synapses in the cerebellum and on inhibitory synapses formed by parvalbumin- or somatostatin-positive neurons in the cerebellum, hippocampus, and medial prefrontal cortex. Our results show that pan-neurexin deletions produce dramatically different phenotypes in synapses, ranging from modest to massive impairments in synapse assembly (climbing-fiber and parvalbumin-positive synapses) to severe but selective decreases in presynaptic action potential-induced Ca^2+^-transients (somatostatin-positive synapses). Thus, neurexins perform powerful but distinct context-dependent roles in different synapses that shape the brain’s circuits.

**Significance Statement:**

Neurexins are abundant presynaptic adhesion molecules expressed from three genes in more than a thousand splice variants. Although neurexins are well studied, no analysis that compares neurexin deletions in multiple types of synapses is currently available. Here, we provide such an analysis by examining triple conditional knockout mice that delete all neurexins except for Neurexin-1γ. Using neuron-specific manipulations combined with slice electrophysiology, two-photon Ca^2+^ imaging and immunohistochemistry, we show that pan-neurexin deletions produce distinct phenotypes in different synapses, ranging from impairments in synapse assembly (climbing-fiber and parvalbumin-positive cortical synapses) to severe selective decreases in presynaptic action potential-induced Ca^2+^- transients (somatostatin-positive cortical synapses). Our data reveal context-dependent distinct functions of neurexins in different synapses whose properties shape the input-output relations of neural circuits.

## Introduction

Neurexins are presynaptic cell-adhesion molecules that were identified as α-latrotoxin receptors (1-4). In vertebrates, three neurexin genes (*Nrxn1, Nrxn2*, and *Nrxn3* in mice) encode longer α- and shorter β-neurexins from independent promoters (3, 5). In addition, the *Nrxn1* gene encodes a third, even shorter γ-neurexin also using an internal promoter (6). Neurexins are evolutionarily conserved with a single α-neurexin expressed in Drosophila (5) and a single α- and γ-neurexin expressed in C. elegans (7). In addition, broadly defined neurexin-related genes, such as CNTNPs, are identifiable throughout the animal kingdom, including in cnidaria that contain only a rudimentary nervous system (8, 9).

**Figure 1.**
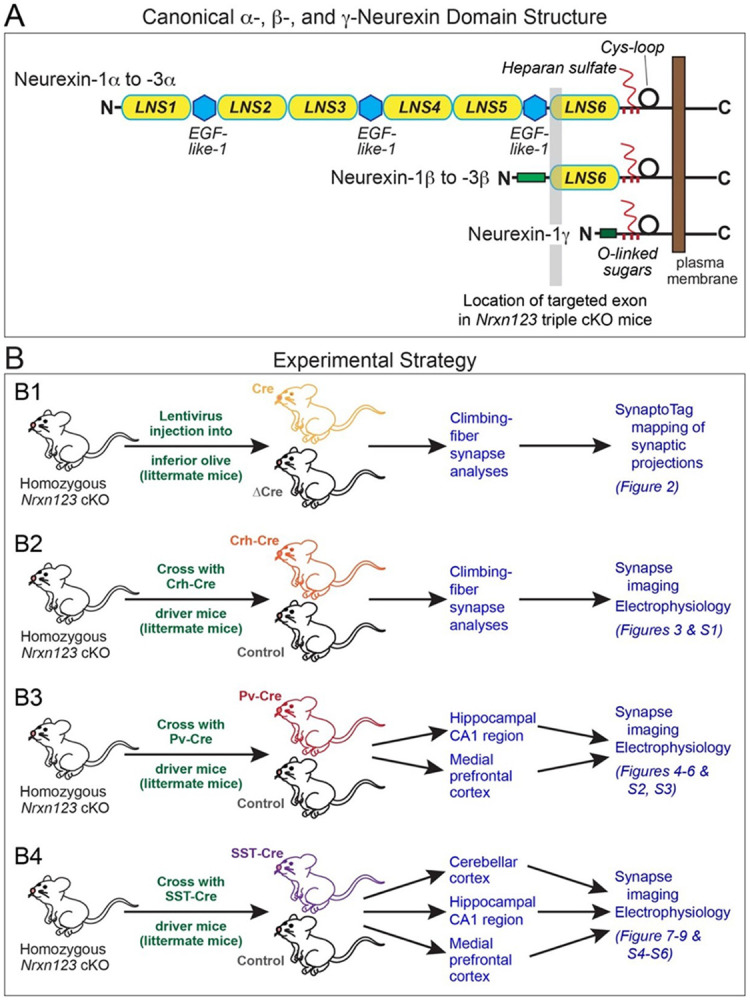
Domain structures of neurexins and experimental design of the present study **A**, Domain structures of neurexins. Three genes (*Nrxn1-3* in mice) encode α- and β-isoforms from independent promoters (1-3), and the Nrxn1 gene additionally encodes a γ-isoform from a third promoter (6). The conditional triple Nrxn123 KO mice used here (referred to as pan-neurexin deletion) abolish expression of all neurexin isoforms except for Nrxn1γ by deleting the first out-of-frame exon shared by α- and β-neurexins. Note that neurexins contain a variable number of LNS- and EGF-like domains and a constant Cys-loop domain followed by a transmembrane region and a cytoplasmic sequence. A subset of neurexins is modified by heparan sulfate, as indicated (10), depending on the expression of FAM19A1-4 and CA10/11 proteins that bind to the Cys-loop domain in the secretory pathway and inhibit the heparan sulfate modification (6, 73). The location of the exon 18 that is floxed in triple *Nrxn123* cKO mice is indicated. **B**, Schematic of the experimental analyses performed for the current study. We used four different mouse models (B1-B4) to analyze 5 different synapses in three brain regions (cerebellar climbing-fiber synapses, hippocampal and cortical synapses formed by parvalbumin-positive (Pv+) or somatostatin-positive (SST+) interneurons). All analyses involved littermate mice whenever possible and were carried out on anonymized samples that blinded the experimenter to the genotype of the sample.

α-Neurexins are composed of a large extracellular sequence comprising six LNS-domains (for laminin/neurexin/sex-specific globulin-domain) with three interspersed EGF-like domains, followed by an O-linked carbohydrate attachment sequence, a single heparansulfate attachment site (10), a cysteine-loop domain (6), a transmembrane region, and a short cytoplasmic sequence (1-3) (Figure 1A). β-Neurexins contain a short β-neurexin-specific N-terminal sequence that then splices into the α-neurexin sequence N-terminal to the last, sixth LNS-domain; from that point on, α- and β-neurexins are identical (3). Neurexin-1γ, finally, also contains a unique short N-terminal sequence that then splices into the α- and β-neurexin sequences C-terminal to the last, sixth LNS-domain, again with identical C-terminal sequences (6) (Figure 1A).

Individual neurexins are essential for multiple synaptic functions in C. elegans, Drosophila, mouse, and human neurons, but no uniform overall role for neurexins has emerged (7, 11-41). Genetic mutations of neurexin genes, analyzed in various animals and different synapses, uncovered diverse phenotypes that affect multiple aspects of synaptic function. In mice, for example, constitutive triple KO of all α-neurexins is lethal (11). Several studies showed that triple α-neurexin KO brains at birth exhibited a modest decrease in inhibitory but not excitatory synapse density and displayed dramatic impairments in excitatory and inhibitory synaptic transmission due, at least in part, to a loss in presynaptic Ca^2+^-influx without affecting axon or dendrite outgrowth (11, 32, 33, 42, 43). Moreover, the triple α-neurexin KO mice exhibited an impairment in neuropeptide signaling (44).

Triple β-neurexin KO mice, conversely, also display a survival phenotype, albeit a milder one than that of triple α-neurexin KO mice, consistent with the relatively lower expression of β-neurexins than α-neurexins (21, 45). Furthermore, triple β-neurexin KO mice exhibit a synaptic transmission impairment that is due, at least in part, to a disinhibition of tonic endocannabinoid signaling (21) and a robust decrease in the number of presynaptic dense-core vesicles, again suggesting functions in neuropeptide signaling (46).

Triple α/β-neurexin KO mice that target both α- and β-neurexins, finally, were reported by us in 2017 in a paper that we retracted because four images contained inexplicable microduplications that did not materially affect the conclusions of the paper (47, 48). The images could not be corrected because the original samples were no longer retrievable after a decade, although other raw data were fully available. The Chen et al. (2017) paper (47) was foundational because it demonstrated that the triple α/β-neurexin deletions (called pan-neurexin deletions) produced distinct phenotypes in different synapses without abolishing synapse formation, suggesting that neurexins are not essential for synapse formation as such but shape synapses in a context-specific manner. This conclusion was expanded in subsequent studies showing among others that, in excitatory Calyx of Held synapses and in synapses formed by MNTB neurons on the superior olive, the conditional triple α/β-neurexin KO produced a selective loss of Ca^2+^ influx (49, 50). Additional studies reported that the conditional triple α/β-neurexin KO induces a general disorganization of presynaptic active zones that includes impairments in presynaptic receptors (51), results in a decrease in Ca^2+^-triggered exocytosis in dopaminergic neurons (52), and may cause neuronal cell death in olfactory sensory neurons, cerebellar granule cells, and serotonergic neurons of the brain stem (53-55).

Thus, present analyses of the global functions of neurexins suggest important and robust synaptic and possibly non-synaptic roles in many different types of neurons. However, no direct comparison of α/β-neurexin KO-induced phenotypes in different types of neurons and synapses is currently available after retraction of our original study (47). Moreover, in many studies the phenotypes were examined primarily morphologically without functional analyses of synaptic transmission, rendering the nature of observed changes unclear. To address these knowledge gaps at least in part, we here aimed to specifically clarify whether neurexins perform canonical, broad functions in all synapses or synapse-specific functions that depend on the pre- and postsynaptic neuron types. For this purpose we reanalyzed the raw data of our retracted Chen et al. (2017) paper (47) and performed additional experiments to complement the original available data, with all raw data made publicly available for independent assessments (website link: https://purl.stanford.edu/xp149zq3345). As a result, the present study reports an electrophysiological and morphological analysis of the effect of the triple α/β-neurexin conditional deletion on multiple well-defined synapses, namely excitatory climbing-fiber synapses in the cerebellum and inhibitory synapses formed by parvalbumin-positive (Pv^+^) or somatostatin-positive (SST^+^) interneurons on hippocampal CA1 neurons and on layer 5 pyramidal neurons in the medial prefrontal cortex (mPFC). Our data reveal that the triple presynaptic neurexin deletions produce two distinct principal types of phenotypes in the examined synapses, namely a loss of synapses or a decrease in voltage-gated presynaptic Ca^2+^ influx, both of which result in a decrease in synaptic connectivity. Thus, our results indicate that neurexins perform functionally diverse fundamental roles in different types of synapses.

## Results

### Design and objective.

The primary goal of the present study is to compare the effects of a deletion of all neurexins across different, well-defined types of synapses in multiple brain regions. For this purpose, we quantitatively reanalyzed the existing raw data of our previous paper (47) that we retracted because of discrete image abnormalities (48) and performed additional experiments to complement the previously obtained data (see Experimental Design, Figure 1B), with public posting of all raw data for access to the community (SDR weblink: https://purl.stanford.edu/xp149zq3345). This effort was made to ensure that the results from our original foundational study (47) would not be lost for the scientific community and that the conclusions of this paper could be validated by new independent analyses.

### Presynaptic neurexin deletion from climbing-fiber synapses modestly impairs synapse assembly.

**Figure 2.**
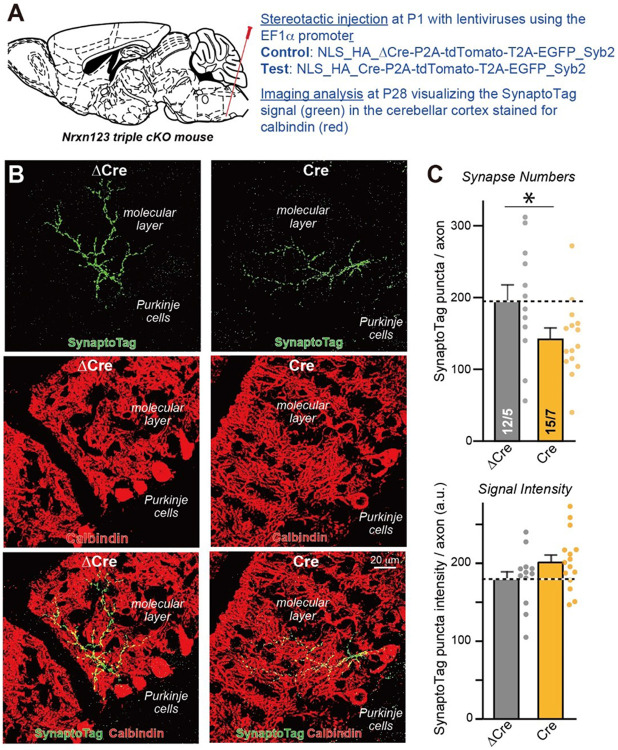
Sparse pan-neurexin deletions in the inferior olive by lentiviral expression of Cre recombinase produce a significant decrease in synapse density as measured by SynaptoTag tracing **A**, Schematic of the stereotactic ablation of neurexins in the inferior olive by lentiviral expression of wild-type active (Cre) and mutant inactive (ΔCre) Cre-recombinase. **B**, Representative images of the cerebellar cortex from injected mice visualizing Purkinje cells by calbindin immunofluorescence (red) and synaptic specializations formed by infected inferior olive neurons by SynaptoTag labeling (green, Synaptobrevin 2-EGFP). **C**, Quantification of the climbing fiber synapse numbers per climbing fiber (top) and SynaptoTag signal intensity (bottom) at P28 (graphs show means ± SEMs; n = 12/5 and 15/7 climbing fibers/mice for ΔCre and Cre conditions, respectively; * = p<0.05 by Student’s t-test).

To analyze the effect of deleting neurexins from climbing-fiber synapses, we pursued two complementary strategies: Injection of lentiviruses co-expressing Synaptobrevin2-EGFP (SynaptoTag) tracers (56) with mutant (ΔCre, control) or wild-type Cre-recombinase into the inferior olive of newborn *Nrxn123* triple cKO mice (Figure 1, B1; Figure 2A), and crosses of *Nrxn123* triple cKO mice with Crh-Cre driver mice that selectively express Cre-recombinase in inferior olive neurons (57) (Figure 1, B2).

In the first approach, we administered lentiviruses by stereotactic delivery into the inferior olive of newborn mice and analyzed the mice at P28 (Figure 2A). The lentiviruses infected a subset of olivary neurons, resulting in sparse expression of SynaptoTag in a minority of climbing fibers that were identified in the cerebellar cortex by virtue of their SynaptoTag fluorescence (Figure 2B). Deletion of neurexins did not produce an apparent decrease in climbing-fiber numbers but caused a modest, albeit significant decrease (~30%) in climbing-fiber synapses on proximal dendrites of cerebellar Purkinje cells (Figure 2C).

The phenotype of the neurexin deletions we observed here with the viral Cre expression in the inferior olive of triple *Nrxn123* cKO mice was less severe than the phenotype we described originally, which was also for a virally induced neurexin ablation in the inferior olive (47). Notably, the two sets of experiments involved distinct viral approaches. It is possible that the differences were due to viral toxicity in the earlier experiments, since the present experiments were performed with less pervasive viral infections. Alternatively, the present viral infections might have been less effective than the original infections in deleting neurexins. Thus, to independently assess the effect of pan-neurexin deletions in the inferior olive, we used a second, non-viral approach that analyzed littermate pan-neurexin cKO mice containing or lacking a Crh-Cre driver allele, which expresses Cre-recombinase in inferior olive neurons (57).

**Figure 3.**
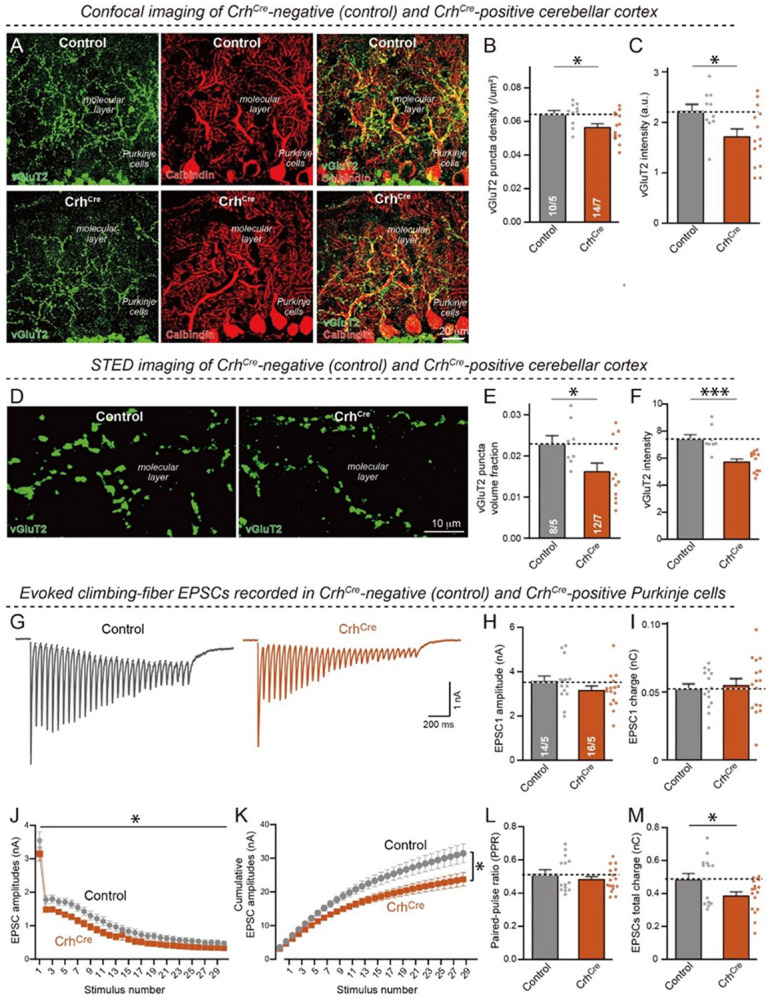
Global pan-neurexin deletions in the inferior olive by crossing *Nrxn123* triple cKO mice with Crh-Cre driver mice modestly but significantly impair climbing-fiber synapses **A-C**, Analysis by confocal microscopy at P28 of the effect of the pan-neurexin deletion in the inferior olive mediated by the Crh-Cre driver line crossed with the triple *Nrxn123* Cko mice (**A**, representative cerebellar cortex sections stained for vGluT2 that is specific for climbing-fiber synapses in the cerebellar cortex (green) and for calbindin (red); **B** & **C**, quantifications of the density (B) and staining intensity (C) of vGluT2-positive climbing-fiber synapses). **D-F**, Analysis by STED super-resolution microscopy of the effect of the Crh-Cre mediated pan-neurexin deletion in the inferior olive on vGluT2-positive climbing-fiber synapses (**D**, representative STED images of cerebellar cortex sections stained for vGluT2 (green); **E** & **F**, quantifications of the vGluT2 puncta volume fraction, used as a proxy for synapse density (E), and of the vGluT2-staining intensity of climbing-fiber synapses (F)). **G**, Representative traces of climbing-fiber EPSCs following a 20-Hz stimulation recorded from Purkinje cells in acute cerebellar slices of triple *Nrxn123* cKO mice lacking or harboring a Crh-Cre allele. Recordings were performed at P33-37 in the presence of PTX and a low dose of CNQX. **H** & **I**, Quantifications of EPSC1 amplitude (H) and EPSC1 charge transfer (I). **J** & **K**, EPSC amplitudes (J) and cumulative EPSC amplitudes (K) plotted as a function of stimulus number. **L** & **M**, Quantifications of the paired-pulse ratio (PPR) (L) and total charge transfer of EPSCs (M). All numerical data are means ± SEMs (n’s = sections/mice or neurons/mice are indicated in the bar graphs); statistical evaluations were performed by Student’s t-test with * = p<0.05; *** = p<0.001). For additional data, see Figure S1.

With the second approach, analysis of the effect of the pan-neurexin deletions in inferior olive neurons on climbing-fiber synapses using immunohistochemistry for the climbing-fiber-specific marker vGluT2 (58) confirmed the modest decrease in synaptic puncta numbers observed with the viral pan-neurexin deletions (Figure 3A-C). This conclusion was further validated by quantitative STED super-resolution microscopy, which reproduced the ~30% loss of synapses caused by the *Nrxn123* triple deletion. With both types of imaging, the remaining synapses exhibited a significant decrease in the vGluT2 staining intensity (~20-30%) (Figure 3D-F).

The expression of Cre-recombinase in all inferior olive neurons in Crh-Cre driver mice enabled additional analyses of the effects of the neurexin deletions by electrophysiology (Figure 3G-M). Consistent with the morphological results, the *Nrxn123* triple deletion produced a decrease in synaptic connectivity, with a non-significant trend towards a decline in the amplitude of single evoked EPSCs that became significant upon analyses of trains of EPSCs induced at 20 Hz (Figure 3G-M; Figure S1). Viewed together, these data uncover a reproducible but modest impairment of climbing-fiber synapse assembly upon deletion of neurexins from inferior olive neurons. This phenotype is similar to, but less severe than, the previously observed phenotype (47), possibly because the previous experiments suffered from viral toxicity or because the present experiments may not have induced as complete as neurexin deletion as the previous experiments.

### The pan-neurexin deletion impairs the organization of inhibitory synapses formed by hippocampal Pv^+^ interneurons.

**Figure 4.**
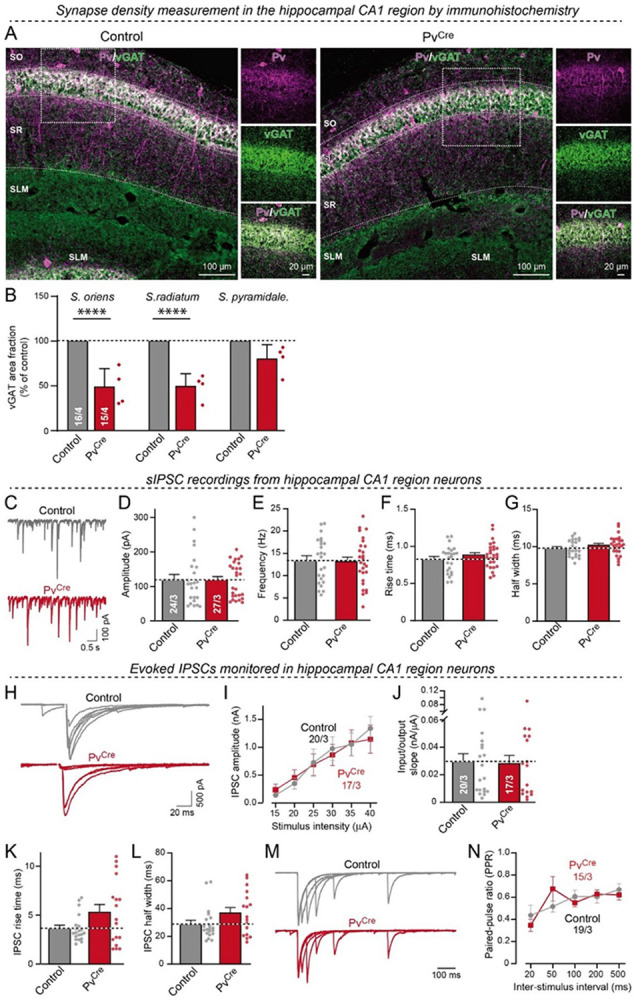
Pan-neurexin deletions in Pv^+^ interneurons of the hippocampal CA1 region cause an apparent decrease in inhibitory synapse numbers but no detectable electrophysiological phenotype when inhibitory neurons are analyzed by extracellular stimulation **A** & **B**, Analysis of the effect of the pan-neurexin deletion in Pv^+^ neurons on the apparent density of inhibitory synapses in the hippocampal CA1 region. Cryostat sections from 8-week-old littermate triple *Nrxn123* cKO mice harboring or lacking the Pv-Cre driver allele were analyzed by immunohistochemistry (**A**, representative confocal images of the CA1 region of the hippocampus immunostained for vGAT (green) and parvalbumin (magenta) to visualize inhibitory synapses formed on CA1 pyramidal neurons. Zoomed in confocal images are represented on the right. **B**, quantifications of the area fraction occupied by inhibitory (vGAT) synapses at CA1 radial layers: SO (Stratum oriens), SR (Stratum radiatum), SP (Stratum pyramidale)). **C**, Representative spontaneous IPSC (sIPSC) traces recorded from CA1 pyramidal neurons in acute hippocampal slices of triple Nrxn123 cKO mice lacking or harboring a PV-Cre allele. **D-G**, Summary graphs of sIPSC amplitude (D), frequency (E), rise time (F) and half-width (G). **H**, Representative evoked IPSC traces recorded from CA1 pyramidal neurons in acute hippocampal slices of triple *Nrxn123* cKO mice lacking or harboring a PV-Cre allele. **I** & **J**, Summary plot of evoked IPSCs as a function of the stimulus intensity and summary graph of the slope of input-output curves of evoked IPSCs. **K** & **L**, Summary graphs of evoked IPSC rise time (K) and half-width (K). **M** & **N**, Representative traces of IPSC PPRs recorded from CA1 pyramidal neurons in acute hippocampal slices of triple *Nrxn123* cKO mice lacking or harboring a PV-Cre allele at different inter-stimulus intervals (M) and summary graph of PPRs as a function of the inter-stimulus interval (N). All numerical data are means ± SEMs (n’s = sections/mice or neurons/mice are indicated in the graphs; statistical evaluations were performed by Student’s t-test (B, D-G, J-L) or two-way ANOVA (I, N) with **** = p<0.0001).

To analyze the effect of the pan-neurexin deletion on a defined inhibitory synapse, we crossed *Nrxn123* triple cKO mice with Pv-Cre mice that express Cre-recombinase under the parvalbumin promoter. We then cut hippocampal sections from 8-week-old littermate *Nrxn123* cKO/Pv-Cre and *Nrxn123* cKO mice, and analyzed the sections by immunohistochemistry for vGAT as a marker of inhibitory synapses and for parvalbumin as a marker of the targeted subset of inhibitory interneurons (Figure 1C; Figure 4A). Since confocal imaging could not resolve individual synaptic puncta in the stained sections, we quantified the overall synapse density as the area fraction occupied by vGAT fluorescence (Figure 4B). These experiments showed that the pan-neurexin deletion in Pv^+^ neurons produced a ~50% decrease of the vGAT signal in the S. oriens and S. radiatum of the CA1 region and a lesser ~20% decrease in the S. pyramidale (Figure 4B). Given that the signal for synaptic markers observed by immunohistochemistry depends on thresholding, the decrease in synaptic signal observed here would be consistent with a decrease in synapse numbers or with a synapse assembly impairment that alters the size of synapses without a loss of synapse numbers.

We next analyzed inhibitory synapses in the hippocampal CA1 region by electrophysiology of 8-week-old littermate *Nrxn123* cKO/Pv-Cre and *Nrxn123* cKO mice (Figure 4C-N). However, we detected no significant phenotype after the Pv^+^ neuron-specific pan-neurexin deletion using measurements of spontaneous IPSCs (sIPSCs) (Figure 4C-G), input-output curves of evoked IPSCs (Figure 4H-L), and paired-pulse IPSC measurements (Figure 4M, N). This result was unexpected, given the robust decrease in synaptic vGAT signal observed by immunohistochemistry (Figure 4A, B). The observed lack of an electrophysiological phenotype here could be due to several factors since we are measuring overall inhibitory synaptic transmission in these experiments. Inhibitory synapses on pyramidal neurons from other, Pv^−^ neurons might dominate and a subset of Pv^+^ neurons might form synapses on other inhibitory neurons (59) that in turn innervate pyramidal neurons, causing a disinhibition of inhibitory responses on pyramidal cells when Pv^+^ neuron synapses are impaired. Thus, to obtain a clearer analysis, paired recordings are required, which motivated us to analyze the mPFC where paired recordings are more straightforward.

### The pan-neurexin deletion decreases assembly of inhibitory Pv^+^ interneuron synapses on pyramidal neurons in the mPFC.

**Figure 5.**
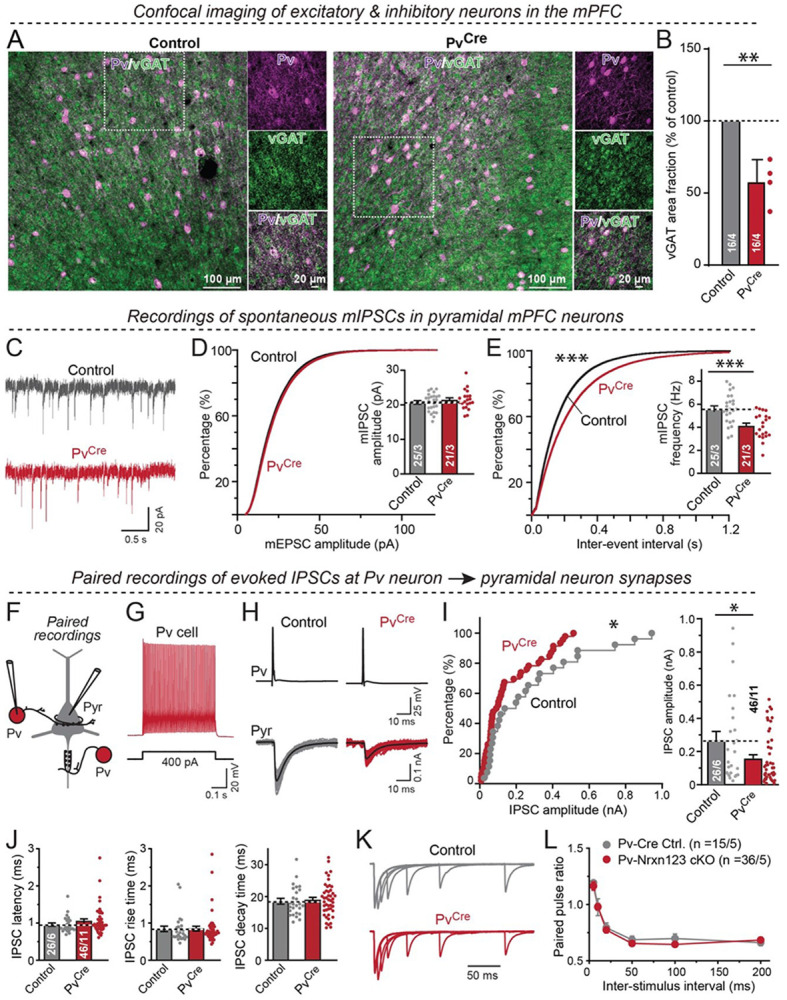
Paired recordings reveal that pan-neurexin deletions in Pv^+^ interneurons decrease inhibitory synapse numbers and synaptic transmission at pyramidal neuron synapses of the mPFC **(A)** Analysis of the effect of the pan-neurexin deletion in Pv^+^ neurons on the apparent density of inhibitory synapses in the medial prefrontal cortex (mPFC). Cryostat sections from littermate 8-week-old triple *Nrxn123* cKO mice harboring or lacking the Pv-Cre driver allele were analyzed by immunohistochemistry for vGAT (green) and parvalbumin (magenta) to visualize inhibitory synapses formed on mPFC pyramidal neurons. Zoomed in confocal images are represented at the bottom. **(B)** Quantifications of the area fraction occupied by inhibitory synapses identified by vGAT staining. **(C)** Representative mIPSC traces recorded from mPFC layer 5 pyramidal neurons in acute mPFC slices from littermate control *Nrxn123* cKO and *Nrxn123* cKO/ Pv-Cre cells. **(D** & **E)** Cumulative distribution plots of mIPSC amplitudes (left) and inter-event intervals (right) monitored in pyramidal neurons of *Nrxn123* cKO and *Nrxn123* cKO/ Pv-Cre mice (insets = summary graphs of average mIPSC amplitude (left) and frequency (right)). **(F)** Diagram of paired recording to monitor inhibitory synapses formed by Pv^+^ interneurons on the perisomatic region and axon initial segments of pyramidal neurons (Pyr). For all recordings of interneuron-pyramidal neuron pairs, the necessity of visual identification of interneurons (mediated by viral injections of AAV-EF1α-DIO-eYFP) required that controls were from non-littermate Pv-Cre mice with a similar genetic background. The success rate of establishing pairs during paired recordings was similar between *Nrxn123* cKO/ Pv-Cre (52 successes/141 pairs tested (36.8%)) and Pv-Cre mice (32 successes/88 pairs tested (36.3%); Table S1). **(G)** Representative traces of a typical fast-spiking firing pattern of Pv^+^ interneurons. **(H)** Representative traces showing unitary synaptic connections between a Pv^+^ interneuron and a pyramidal neuron in Pv-Cre control and *Nrxn123* cKO/ Pv-Cre (grey traces, overlaid individual responses from different trials; black/blue traces, averaged responses). **(I)** Left, cumulative probability plot of unitary mean IPSC amplitudes (inset = summary graph of IPSC amplitudes) showing that Pv^+^ interneurons from *Nrxn123* cKO/Pv-Cre mice exhibit a decrease in synaptic strength. **(J)** Summary plots of the latency (left), rise time (center), and decay time (right) of unitary IPSCs in Pv-interneuron → pyramidal neuron synapses. **(K)** Representative traces showing PPRs of unitary IPSCs in Pv-interneuron → pyramidal neuron synapses at different inter-stimulus intervals. (**L**) Summary graph of PPRs as a function of the inter-stimulus interval. All numerical data are means ± SEMs (n’s = sections or slices/mice are indicated in the bar graphs); statistical evaluations were performed by Student’s t-test (B-J) or two-way ANOVA followed by Bonferroni *post-hoc* test (L) with * = p<0.05; ** = p<0.01; *** = p<0.001; non-significant comparisons are not labeled. For additional data, see Figure S2.

Our observations in the hippocampus suggested that the pan-neurexin deletion produces an impairment in synapse assembly, but provided unclear results because overall inhibitory synapses, instead of specifically Pv^+^ synapses, were analyzed. To gain specific insight into Pv^+^ inhibitory synapses and investigate another brain region, we examined the effect of the pan-neurexin deletion on Pv^+^ synapses of the mPFC (Figure 1, B3; Figure 5). Immunohistochemistry for vGAT and parvalbumin again uncovered a significant decrease in inhibitory synaptic signal (~50%) as measured as the vGAT-positive area fraction (Figure 5A, B). Moreover, recordings of miniature IPSCs (mIPSCs) from layer 5 pyramidal neurons of the mPFC revealed a ~25% decrease in mIPSC frequency without a significant decrease in mIPSC amplitude (Figure 5C-E; Figure S2), consistent with the decrease in synapse assembly.

Next, we embarked on a mechanistic analysis of synapses formed by Pv^+^ interneurons onto pyramidal neurons using paired recordings in acute slices (Figure 5F). This approach avoids the possibility that is inherent in measurements of overall synaptic responses, namely that the monitored synaptic outputs are produced by a mixture of inhibitory inputs and are influenced by disinhibition. For paired recordings, we stereotactically injected the mPFC of *Nrxn123* cKO/Pv-Cre and Pv-Cre control mice with AAVs expressing double-floxed eYFP at P21 and analyzed the mice at P35-P40. Note that we needed to use genetic labeling of Pv^+^ interneurons in order to perform paired recordings, which made it difficult to record from littermate mice. Therefore, test and control slices for these experiments (and in the related experiments with SST^+^ interneurons described below) were not obtained from littermate mice. Instead, the control mice used in these experiments were generated from *Nrxn123* cKO/Pv-Cre mice by generating Pv-Cre control mice that were separated less than 3 generations from *Nrxn123* cKO/Pv-Cre mice, suggesting that they harbor a similar genetic background. Only age-matched control and test mice were used, which were manipulated in parallel at the same time.

We patched adjacent Pv^+^ interneurons and pyramidal neurons and confirmed the fast-spiking properties of the Pv^+^ interneurons by current injections (Figure 5F, G). We probed for synaptic connections by monitoring monosynaptic IPSCs (Figure 5H). Slices from *Nrxn123* cKO/Pv-Cre and Pv-Cre control mice exhibited a similar rate of success in establishing connected pairs during paired recordings (32 successes/88 pairs tested vs. 52 successes/141 pairs tested, respectively; see Table 1). However, the IPSC amplitudes in Pv^+^ interneuron → pyramidal neuron synapses were decreased by ~50% in Nrxn123 cKO/Pv-Cre mice compared to Pv-Cre mice (Figure 5I), similar to the decrease in the synaptic vGAT signal (Figure 5B). In neurexin-deficient Pv^+^ neurons, high-amplitude synaptic responses were preferentially lost, without a change in IPSC kinetics (Figure 5J). We detected no significant change in paired-pulse ratios, suggesting that the decrease in IPSC amplitude is likely not due to a change in release probability (Figure 5K, L). Moreover, we found no detectable change in IPSC kinetics (Table 1). Overall, these experiments indicate that the pan-neurexin deletion from Pv^+^ interneurons severely impairs the formation of synaptic connections.

### Action potential-induced Ca^2+^ influx into the presynaptic terminals of Pv^+^ interneurons on pyramidal neurons in the mPFC is not impaired by the *Nrxn123* triple deletion.

**Figure 6.**
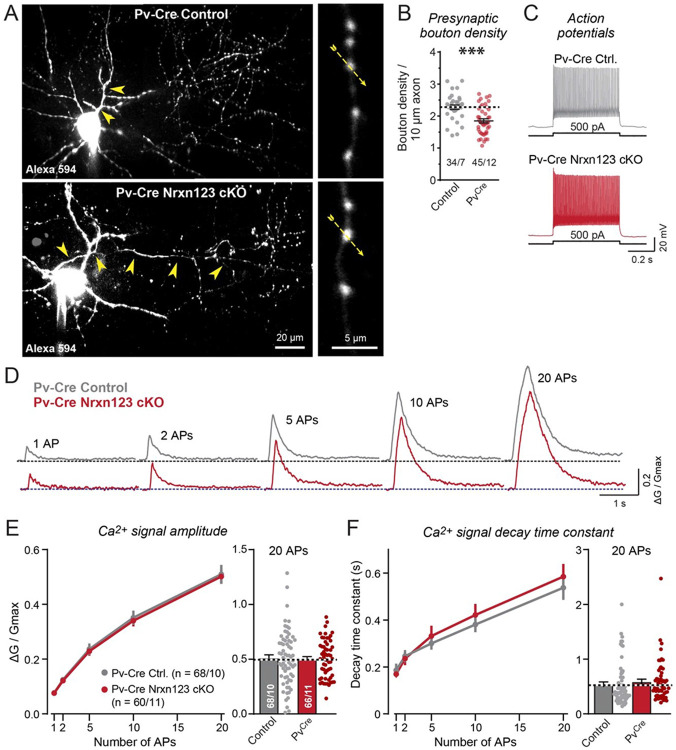
Two-photon microscopy demonstrates that pan-neurexin deletions in Pv^+^ interneurons do not alter action potential-induced Ca^2+^-influx into presynaptic terminals but decrease the density of presynaptic axonal boutons in the mPFC **(A)** Pv^+^ interneurons visualized by Alexa Fluor 594 fluorescence in Pv-Cre control and in *Nrxn123* cKO/ Pv-Cre mice. Neurons identified via virally administered double-floxed mCherry were filled via the patch pipette with Alexa Fluor 594 (50 μM; to visualize the axonal arbor and dendrites) and Fluo-5F (200 μM; to monitor presynaptic Ca^2+^-transients). The left and right images depict representative z-stack projections of the entire neuron (arrowheads = axon) or expanded views of axonal boutons (yellow dashed arrows = line scans during action potential stimulation), respectively. **(B)** Decreased density of axonal boutons of neurexin-deficient Pv^+^ interneurons (circles, individual axon branches; lines, means ± SEM). **(C)** Representative traces depicting the fast-spiking firing pattern of Pv^+^ interneurons in Pv-Cre and *Nrxn123* cKO/ Pv-Cre interneurons. **(D)** Representative traces of Ca^2+^-transients monitored via Fluo-5F fluorescence in presynaptic boutons by line scans as indicated in A. Trains of action potentials (1, 2, 5,10, 20 APs at 50 Hz) were elicited by injecting current pulses (2 nA, 1 ms) via the recording pipettes. **(E)** Left, summary plots of the peak amplitudes (ΔG/Gmax) of Ca^2+^-transients as a function of action potential number monitored in presynaptic boutons of Pv^+^ interneurons. Right, summary of peak amplitudes of Ca^2+^-transients evoked by 20 APs. (**F**) Same as (**E**), but for the decay times, measured as the time required for the signal to decay from 100% to 37% of its peak amplitude. Data in B and E are means ± SEM (numbers indicate number of axon branches/neurons analyzed [B] or boutons/neurons analyzed [E]); statistical comparisons were performed with Student’s t-test (*P<0.05; **P<0.01; ***P<0.001) or two-way ANOVA followed by Bonferroni *post-hoc* test; non-significant comparisons are not labeled.

To further characterize the effect of the *Nrxn123* deletion on Pv^+^ interneuron synapses, we examined their action-potential induced Ca^2+^-influx. We stereotactically injected the mPFC with AAVs expressing double-floxed mCherry at P21 and analyzed the mice at P35-P40. Pv^+^ interneurons, identified in acute slices from *Nrxn123* cKO/Pv-Cre and wild-type Pv-Cre mice by expression of mCherry, were then filled via a patch pipette with Alexa Fluor 594 (50 μM) to visualize the axonal arbor and with Fluo-5F (200 μM) to monitor presynaptic Ca^2+^-transients. Afterwards, we imaged presynaptic terminals by two-photon microscopy (Figure 6A).

Analyses of the density of presynaptic boutons in the axonal arbors of Pv^+^ interneurons revealed that the *Nrxn123* deletion caused a modest but significant decrease in bouton density (~15%) consistent with a decrease in synapse numbers (Figure 6B). We confirmed by test-current injections that the patched neurons were indeed fast-spiking neurons as expected for Pv^+^ interneurons (Figure 6C) and measured presynaptic Ca^2+^-transients as a function of presynaptic action potentials. Using this experimental configuration, we could reliably detect Ca^2+^-transients in response to single action potentials (Figure 6D). In control neurons, we observed a nearly linear relationship between the number of action potentials and Ca^2+^-transients in presynaptic nerve terminals of Pv^+^ neurons, as measured via the Ca^2+^-indicator signal (Figure 6E). Notably, the presynaptic deletion of neurexins caused no change in the amplitude or the decay kinetics of the Ca^2+^-transients, consistent with the lack of a change in paired-pulse ratio.

Viewed together, our results suggest that deletion of presynaptic neurexins from Pv^+^ interneurons impairs the assembly of synapses formed by Pv^+^ neurons on pyramidal neurons and decreases the strength of these synapses without changing their action potential-induced Ca^2+^-influx.

### The *Nrxn123* deletion does not significantly alter inhibitory synapse numbers formed by SST^+^ interneurons in the hippocampus or mPFC.

**Figure 7.**
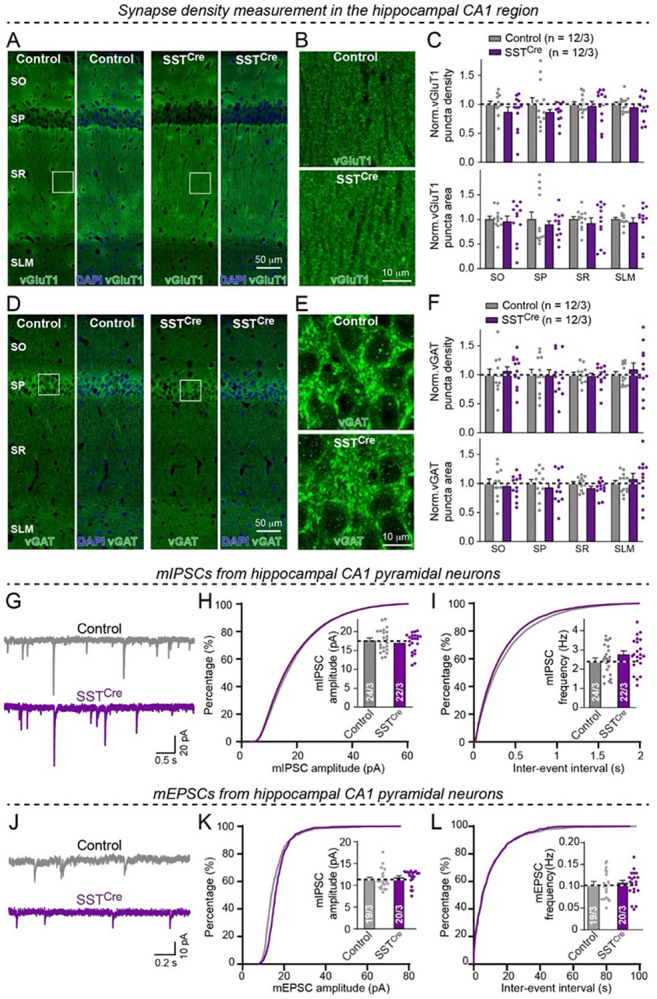
Pan-neurexin deletions in SST^+^ interneurons do not change overall synapse numbers, mIPSCs, or mEPSCs in the CA1 region of the hippocampus **(A-C)** Deletion of all neurexins in SST^+^ interneurons does not change the density and area of vGluT1-positive synaptic puncta in all hippocampal subregions, including the Stratum oriens (SO), Stratum pyramidale (SP), Stratum radiatum (SR), and Stratum lacunosum-moleculare (SLM). **(A)** Representative confocal images of vGluT1 immunostaining showing an overview of the CA1 region from littermate control Nrxn123 cKO (left) and *Nrxn123* cKO/SST-Cre mice (right). **(B)** Higher-magnification views of the boxed regions in (A). (**C**) Summary graphs of vGluT1^+^ puncta density (top) and area (bottom) quantified in each CA1 subregion. (**D-F**) Same as (**A-C**), but for vGAT-positive inhibitory synaptic puncta. (**G-I**) Deletion of all neurexins in SST^+^ interneurons do not change mIPSCs recorded from CA1 pyramidal neurons. (**G**) Representative mIPSC traces. (**H-I**) Cumulative distributions of mIPSC amplitudes (H) and inter-event intervals (I). Insets, summary graphs of mean mIPSC amplitude and frequency. (**J-L**) Same as (**G-I**), but for mEPSCs. All numerical data are means ± SEMs (n’s = sections/mice or neurons/mice are indicated in the bar graphs); statistical evaluations were performed by Student’s t-test, two-way ANOVA followed by Bonferroni *post-hoc* test (C, F) or two-tailed Kolmogorov-Smirnov test (cumulative distribution). For synapse measurements in the cerebellar cortex, see Figure S3.

To test whether neurexins perform a similar function in another type of inhibitory synapse that differs from those of Pv^+^ interneurons, we analyzed synapses formed by SST^+^ interneurons. We used the same strategy as outlined above for Pv^+^ interneurons, except that we employed SST-Cre mice instead of Pv-Cre mice (Figure 1B4). Although SST is expressed in the cerebellum during early postnatal development (60), we detected no change in the density of vGluT1^+^, vGluT2^+^, or vGAT^+^ puncta in cerebellar sections, suggesting that the pan-neurexin deletion in SST^+^ cerebellar neurons does not cause a major change in excitatory or inhibitory synapse densities (Figure S3). A similar observation was made for the hippocampal CA1 region, where the pan-neurexin deletion in SST^+^ neurons again caused no detectable change in the density of vGluT1^+^ or vGAT^+^ puncta (Figure 7A-F). Moreover, when we measured the frequency and amplitude of miniature EPSCs (mEPSCs) and mIPSCs in acute slices from the CA1 region, we detected no significant change as a result of the pan-neurexin deletion in SST^+^ neurons (Figure 7G-L).

**Figure 8.**
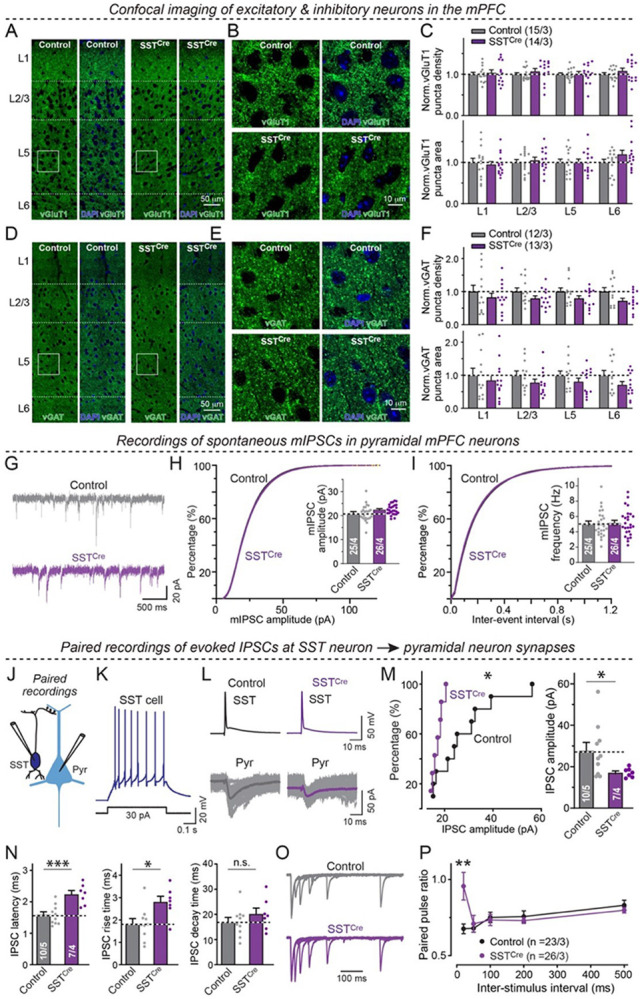
Pan-neurexin deletions in SST^+^ interneurons of the mPFC do not significantly decrease inhibitory synapse numbers but robustly impair evoked synaptic transmission as monitored by paired recordings **(A-C)** Deletion of all neurexins in SST^+^ interneurons does not change the density and area of vGluT1-positive synaptic puncta across all layers of the mPFC. **(A)** Representative confocal images of vGluT1 immunostaining showing mPFC sections from littermate control Nrxn123 cKO (left) and *Nrxn123* cKO/SST-Cre mice (right). Cortical layers (L1–L6) are indicated on the left. **(B)** Higher-magnification views of the boxed regions in (A). (**C**) Summary graphs of vGluT1^+^ puncta density (top) and area (bottom) quantified in each cortical layer. (**D-F**) Same as (**A-C**), but for vGAT-positive inhibitory synaptic puncta. (**G-I**) Deletion of all neurexins in SST^+^ interneurons do not change mIPSCs recorded from layer 5 pyramidal neurons in mPFC. (**G**) Representative mIPSC traces. (**H-I**) Cumulative distributions of mIPSC amplitudes (H) and inter-event intervals (I). Insets, summary graphs of mean mIPSC amplitude and frequency. **(J)** Paired recording configuration to monitor inhibitory synapses formed by SST^+^ interneurons on the distal dendrites of a pyramidal neuron (Pyr). For all recordings of interneuron-pyramidal neuron pairs, the necessity of visual identification of interneurons (mediated by viral injections of AAV-EF1**α**-DIO-eYFP) required that controls are from non-littermate SST-Cre mice with a similar genetic background. **(K)** Representative traces of a typical low-threshold-spike firing pattern of SST^+^ interneurons. **(L)** Representative paired-recording traces showing unitary synaptic connections between an SST^+^ interneuron and a pyramidal neuron in SST-Cre control or *Nrxn123* cKO/SST-Cre mice. SST^+^ cells were identified in slices by eYFP fluorescence after infection with AAVs containing double-floxed eYFP expression cassettes. IPSCs were evoked by current injections into patched presynaptic SST^+^ interneurons (top), and recorded from patched postsynaptic pyramidal neurons (bottom grey traces, overlaid individual responses; black/purple traces, averaged responses). **(M)** Cumulative probability plot of unitary mean IPSC amplitudes (inset = summary graph of IPSC amplitudes) showing that in the overall cell population, the synaptic strength is preferentially decreased in neurons with high IPSC amplitudes. **(N)** Summary plots of latency (left), rise time (center), and decay time (right) of unitary IPSCs in SST-interneuron → pyramidal neuron synapses. **(O-P)** Representative traces showing PPRs of unitary IPSCs in SST-interneuron → pyramidal neuron synapses at increasing inter-stimulus intervals (O), and summary graph of the mean PPRs as a function of the inter-stimulus interval (P). Unitary IPSCs were evoked by optogenetic stimulation of SST^+^ cells after stereotactic injection of the mice with AAVs encoding CAG-DIO-ChR2-tdTomato. Data in summary graphs are means ± SEM; statistical comparisons were performed with Student’s t-test (*P<0.05; **P<0.01; ***P<0.001; non-significant comparisons are not labeled). Numbers indicate the number of cells and mice examined. For additional data, see Fig. S4.

Next, we monitored synapses in the mPFC as a function of the *Nrxn123* deletion in SST^+^ neurons (Figure 8). Again, we detected no significant change in excitatory vGluT1^+^ and in inhibitory vGAT^+^ puncta densities, although there was a trend towards a decrease for the latter (Figure 8A-F). We then measured the mIPSC frequency and amplitude in layer 5 pyramidal neurons and also detected no significant change induced by the *Nrxn123* deletion in SST^+^ neurons (Figure 8G-I).

### The *Nrxn123* deletion significantly decreases the presynaptic release probability in SST^+^ interneurons.

Although in the experiments described above we detected no overall phenotype in inhibitory synapses that was induced by the pan-neurexin deletion in SST^+^ neurons, a phenotype could have been missed since SST+ neurons constitute only a subset of inhibitory neurons and may also be contributing to disinhibitory circuits (61). Thus, we performed paired recordings, comparing *Nrxn123* cKO/SST-Cre with wild-type SST-Cre mice (Figure 8J). Similar to the paired recordings of Pv^+^ wild-type and *Nrxn123* cKO neurons, we detected no significant difference between the connection frequency of SST^+^ wild-type and *Nrxn123* cKO neurons, although there was a trend towards fewer pairs in the *Nrxn123* cKO neurons (7 successes/61 pairs vs. 11 successes/ 43 pairs, respectively; Table 1). Strikingly, we found that the presynaptic deletion of neurexins in SST^+^ neurons caused a large decrease in the IPSC amplitude (~40%; Figure 8L, M). No SST^+^ interneuron → pyramidal neuron synaptic connection exhibited an IPSC amplitude of >20 pA in *Nrxn123* cKO/SST-Cre mice, whereas >50% of these synaptic connections exhibited IPSCs with amplitudes of >20 pA in wild-type mice (Figure 8L, M). In addition, IPSCs elicited by stimulation of neurexin-deficient SST^+^ interneurons were slower, as indicated by an increased synaptic latency (~30%) and increased rise time (~30%) (Figure 8N; Table 1).

To test whether the decrease in synaptic strength in SST^+^ interneuron synapses is due to a decrease in release probability, we measured the paired-pulse ratio at SST^+^ interneuron → pyramidal neuron synapses. For this purpose, the low synaptic strength of individual connections made it necessary to simultaneously stimulate multiple synaptic inputs from SST^+^ interneurons onto the same postsynaptic neuron. We achieved this by stereotactically infecting the mPFC of Nrxn123 cKO/SST-Cre and wild-type SST-Cre mice at P21 with AAVs that encode a double-floxed expression cassette for a fusion protein composed of channelrhodopsin-2 and tdTomato (62). We then recorded IPSCs evoked by light stimulation from pyramidal neurons in layer 5 of the mPFC at P35-P40. The stimulation intensity was adjusted until a typical monosynaptic response with linear rise and exponential decay times was obtained. Under these conditions, the first IPSC usually had an amplitude of 0.1-0.2 nA. Although this experimental setup does not allow measurements of synaptic strength, it enables the administration of two closely spaced stimuli at defined intervals to monitor relative IPSC amplitudes during paired-pulse ratio measurements. Using this approach, we observed a large increase in paired-pulse ratios selective for short interstimulus intervals (Figure 8O, P). Together with the slower IPSC kinetics, these findings indicate that pan-neurexin deletion in SST^+^ interneurons reduces the presynaptic release probability.

### The *Nrxn123* deletion in SST^+^ interneurons significantly impairs action potential-induced Ca^2+^ influx.

**Figure 9.**
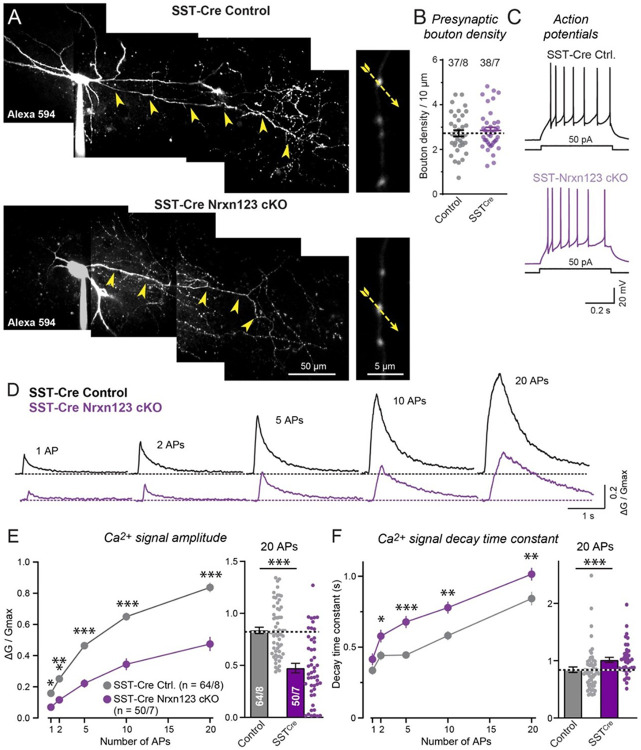
Two-photon microscopy reveals that the pan-neurexin deletion in SST^+^ interneurons in the mPFC suppresses action potential-evoked presynaptic Ca^2+^-transients **(A)** Morphology of SST^+^ interneurons visualized by Alexa Fluor 594 fluorescence in control SST-Cre and in *Nrxn123* cKO/SST-Cre mice. Neurons identified via virally administered double-floxed eYFP were filled via the patch pipette with Alexa Fluor 594 (50 mM; to visualize the axonal arbor) and Fluo-5F (200 μM; to monitor presynaptic Ca^2+^-transients). The top image depicts a representative z-stack projection of the entire neuron (arrowheads = axon). The bottom image displays an expanded view of axonal boutons (yellow dashed arrows = line scans during action potential stimulation). Note that axons of SST^+^ interneurons extend into superficial mPFC layers (~ 500 μm away from the soma). **(B)** Normal density of axonal boutons of neurexin-deficient SST^+^ interneurons (circles, individual cells; lines, means ± SEM). **(C)** Representative traces depicting the low-threshold spiking firing pattern of SST^+^ interneurons in SST-Cre control and *Nrxn123* cKO/SST-Cre interneurons. **(D)** Representative traces of Ca^2+^-transients monitored via Fluo-5F fluorescence in presynaptic boutons by line scans as indicated in A. Trains of APs (1, 2, 5,10, 20 APs at 50 Hz) were elicited by injecting current pulses (2 nA, 1 ms) through recording pipettes. **(E)** Summary plots of the peak amplitudes (ΔG/Gmax) of Ca^2+^-transients as a function of action potential numbers monitored in presynaptic boutons from SST^+^ control and neurexin-deficient interneurons. Right, summary of peak amplitudes of Ca^2+^-transients evoked by 20 APs. (**F**) Same as (**E**), but for the decay times, measured as the time required for the signal to decay from 100% to 37% of its peak amplitude. Data in B and E are means ± SEM (numbers indicate number of axon branches/neurons analyzed [B] or boutons/neurons analyzed [E]); statistical comparisons were performed with Student’s t-test (*P<0.05; **P<0.01; ***P<0.001); non-significant comparisons are not labeled. All numerical data are means ± SEMs (numbers indicate number of axon branches/neurons analyzed [B] or boutons/neurons analyzed [E]); statistical evaluations were performed by Student’s t-test or two-way ANOVA followed by Bonferroni *post-hoc* test with * = p<0.05; *** = p<0.001.

The decrease in release probability that is induced by the neurexin deletion in synapses formed by SST^+^ interneurons in the cortex resembles the phenotype of the pan-neurexin deletion in the calyx of Held synapse, which is caused by a decrease in action potential-triggered Ca^2+^-influx (49). Thus, we tested whether action potential-induced Ca^2+^-influx is decreased by the *Nrxn123* deletion in SST^+^ interneurons (Figure 9A). For this purpose, we monitored the Ca^2+^-dynamics of presynaptic terminals in SST^+^ interneurons, using the same approach as described above for Pv^+^ interneurons. Consistent with the lack of a change in synapse density, we detected no change in the density of presynaptic boutons as a result of the neurexin deletion (Figure 9B). SST^+^ interneurons from *Nrxn123* cKO/SST-Cre and SST-Cre control mice exhibited a typical low-threshold spike firing pattern (Figure 9C). Ca^2+^-transients induced by single action potentials could be reliably identified in individual presynaptic boutons (Figure 9D).

Importantly, we observed that the neurexin deletion produced a large decrease in the amplitude of action-potential-induced Ca^2+^-transients (Figure 9D, E). In control SST^+^ neurons, the Ca^2+^-transient increased almost linearly as a function of action potential numbers. In neurexin-deficient SST^+^ interneurons, the Ca^2+^-transient amplitude was uniformly decreased by ~50%, and the Ca^2+^-transient decay time was significantly increased (Figure 9D-F). These changes account for the decrease in release probability, suggesting that the neurexin deletion decreases synaptic strength in SST^+^ interneuron synapses at least in part by lowering Ca^2+^-influx.

### Pv^+^ and SST^+^ interneurons do not exhibit major differences in the expression of neurexins and other synaptic cell-adhesion molecules as analyzed by single-cell quantitative RT-PCR.

**Figure 10.**
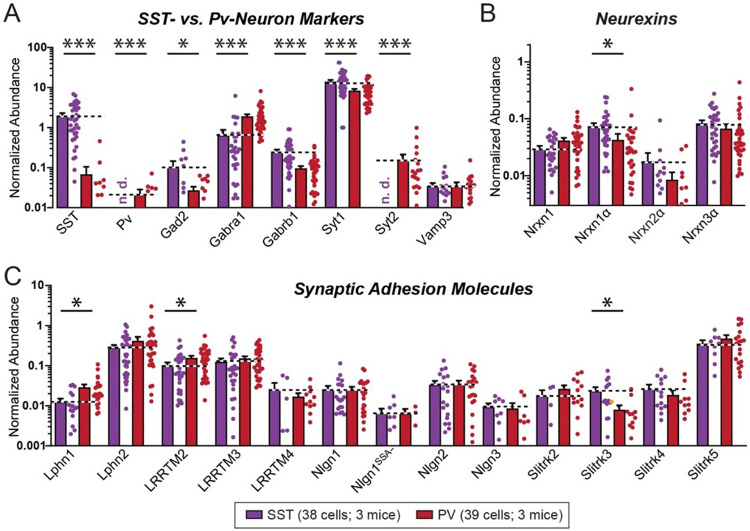
Quantification of mRNA expression levels in Pv^+^ or SST^+^ interneurons from the mPFC fails to identify dramatic differences in the expression of neurexins or other synaptic adhesion molecules **(A)** Average single-cell expression levels of the indicated markers in Pv^+^- and SST^+^- interneurons. Pv^+^- and SST^+^-interneurons were identified in acute slices at P35-40 via their eYFP fluorescence after P21 stereotactic viral injections of AAV-EF1α-DIO-eYFP into Pv-Cre and SST-Cre mice, respectively. Individual cells were patched, cytosol was aspirated, and mRNAs were quantified by qRT-PCR and their normalized abundance calculated as described (45). **(B)** Same as A, but for mRNAs encoding the three principal α-neurexins. **(C)** Same as A, but for the indicated synaptic adhesion molecules other than neurexins. Data are means ± SEM (n = 38/3 and 39/3 cells/mice for SST^+^ and Pv^+^ neurons, respectively; note that for some genes, n’s are lower due to poor quality qRT-PCR reads). Statistical evaluations were performed by Student’s t-test with * = p<0.05; *** = p<0.001. The Student’s t-test is appropriate here since the qRT-PCR assay for each gene is a separate experiment with separate reagents. Only comparisons of relative levels for individual assays are statistically comparable.

A possible explanation for the dramatically different phenotypes of the pan-neurexin deletions in Pv^+^ vs. SST^+^ interneurons of the mPFC is that these neurons might selectively express different neurexins. To test this hypothesis, we compared the relative expression profiles of neurexins in inhibitory Pv^+^ and SST^+^ interneurons of the mPFC using Fluidigm-based quantitative single-cell RT-PCR of cytoplasm aspirated from the neuronal cell bodies via a patch pipette. Specifically, we identified Pv^+^ and SST^+^ interneurons in acute slices from wild-type Pv-Cre or SST-Cre mice injected with AAVs expressing double-floxed eYFP at P21 and analyzed at P35-P40. We patched the labeled neurons, aspirated cytosol, and performed mRNA measurements on the cytosol (Figure 10). We found that apart from typical markers for Pv^+^ and SST^+^ interneurons (such as parvalbumin, somatostatin, and synaptotagmin-2 (63)), both types of interneurons expressed similar overall levels of neurexins and of other synaptic cell-adhesion molecules (Figure 10). Lphn1 and Slitrk3 exhibited modest differences between the two types of interneurons, but all three neurexin genes were expressed similarly. Given the small number of synaptic adhesion molecules surveyed here, our results do not exclude the likelihood that the distinct functions of neurexins in Pv^+^ and SST^+^ neurons are conditioned by the differential expression of synaptic adhesion molecules that remain to be identified.

## Discussion

A large number of studies attest to the physiological importance of neurexins, showing that mutations or deletions of neurexins in organisms ranging from worms to humans cause synaptic dysfunction (37-41). Moreover, alterations in neurexin genes, in particular *NRXN1*, are associated with diverse neuropsychiatric disorders, including autism, schizophrenia, and Tourette syndrome, and anti-neurexin antibodies are prominent in autoimmune encephalitis (64-69). Thus, with thousands of papers listed in PubMed, neurexins are deeply researched. Nevertheless, considerable uncertainty exists regarding the overall and specific functions of neurexins. The physiological consequences of neurexin mutations differ dramatically between experimental preparations and organisms, including the original observation that deletion of all α-neurexins severely impairs mouse survival and synaptic transmission (11) and more recent findings that *Nrxn3* deletions decrease the presynaptic release probability at inhibitory synapses in olfactory bulb neurons (20, 27) and that alternative splicing of *Nrxn1* and *Nrxn3* at splice-site 4 controls postsynaptic NMDARs and AMPARs, respectively, at excitatory synapses (19, 23-25).

A decade ago, we published a study aiming to compare fundamental neurexin functions between diverse synapses by conditionally deleting all neurexins (except for Nrxn1γ) in different types of neurons (47). This pivotal study established that pan-neurexin deletions produced distinct phenotypes in different synapses, a result that was amplified in subsequent papers by examining other types of neurons with similar approaches (49, 50, 52-55). However, we recently retracted the foundational Chen et al. (2017) paper (47, 48) because AI-based image analyses discovered microduplications of small image areas in three panels of the paper, even though these image abnormalities had no significant impact on the paper’s conclusions. As a result, the finding of distinct neurexin functions in diverse synapses was removed from the scientific community, motivating the present study. Here, using a complete reanalysis of the existing raw data from the original study and newly performed complementary experiments, we affirm and expand the original paper’s results. Moreover, to ensure that future AI-based analyses do not detect any anomalies in published images, we are making all raw data publicly available (SDR: https://purl.stanford.edu/xp149zq3345). Our present findings confirm, with some additions and alterations, the original paper’s findings and extend them to additional synapses, affirming the overall conclusion that neurexins are universal regulators of synapse assembly that differentially impact diverse synapses.

Our results establish two major observations. First, in excitatory climbing-fiber synapses and inhibitory hippocampal cortical Pv^+^ neuron→pyramidal neuron synapses, the pan-neurexin deletion produces a partial impairment in synapse assembly, leading to synapse loss (Figure 2-6). Most illuminating here are our paired recordings in the mPFC that revealed that the pan-neurexin deletion decreased the density of synaptic boutons measured in the Ca^2+^-imaging experiments by ~20% (Figure 6B) and reduced the IPSC amplitudes of Pv^+^ interneuron →pyramidal neuron synapses by ~50% (Figure 5I) without affecting the Ca^2+^-dynamics of presynaptic Pv^+^ nerve terminals (Figure 6D-F). Thus, the pan-neurexin deletion likely primarily impairs synapse assembly by rendering synapses smaller and weaker, with a secondary loss of synapses. We observed a similar loss of synapses in the hippocampus but could not detect a change in synaptic transmission here, probably because we measured all synaptic responses in slices using extracellular stimulation instead of paired recordings (Figure 4).

Second, the pan-neurexin deletion severely impaired the function of synapses formed by SST^+^ interneurons without significantly changing synapse numbers (Figures 7-9). The functional impairment of the SST^+^ interneuron synapses supports an essential role for neurexins in enabling the localization of Ca^2+^-channels at synaptic release sites, as described originally for the triple α-neurexin KO mice (11) and previously also confirmed for pan-neurexin KO mice in brainstem synapses (49, 50). This phenotype does not necessarily imply a direct role of neurexins in Ca^2+^-channel recruitment, a role performed by RIMs and RIM-BPs (70, 71), but could reflect an indirect effect. It is striking that none of the three pan-neurexin KO phenotypes described here seems similar to the neurexin-3 cKO phenotypes we described in other inhibitory synapses (20, 27), suggesting that we would probably discover a much larger range of phenotypes if we examined other types of synapses.

Multiple questions arise from our experiments.

1) How can we explain the differences in phenotypes observed in different types of synapses? A plausible hypothesis is that the balance of different neurexins, their various splice variants, and their multifarious pre- and postsynaptic ligands, and not the absolute amounts of these proteins, dictate synaptic functions (45). Although we detected no major differences in the expression of neurexins between Pv^+^ and SST^+^ neurons (Figure 10), differences in alternative splicing of neurexins have large effects on at least some synapse properties (19, 23-25, 27). As a result, presynaptic neurexins might mediate distinct functions even in synapses formed by the same presynaptic neurons onto different postsynaptic cells, depending on the ligand expression of these cells. This may be a general principle of synaptic adhesion molecules. Alternatively, it is conceivable that canonical neurexin functions are differentially compensated in various types of synapses by neurexin-independent pathways. This possibility is enabled by the chronic nature of our cortical manipulations, which provides time for distinct compensatory processes in different types of neurons. As a third, also unlikely possibility given the known properties and localization of neurexins, it is conceivable that the pan-neurexin deletions caused distinct phenotypes in different types of neurons because they impair a common upstream process that is transformed into distinct downstream consequences in various types of synapses. Future experiments studying specific neurexin functions in depth will have to address these questions.

2) Why isn’t the phenotype more severe? In the present analysis, two major types of general phenotypes were observed: a decrease in synapse numbers and an impairment in action potential-triggered Ca^2+^ influx. However, in analyses of manipulations of single neurexins or neurexin splice variants, occasionally more extensive phenotypes were found. For example, constitutive expression of the SS4+ variant of *Nrxn3* in CA1 region neurons causes a large (>50%) decrease in postsynaptic AMPARs at CA1→subiculum synapses (19), whereas deletion of Nrxn3 from inhibitory granule cells in the olfactory bulb greatly decreases (>50%) the presynaptic release probability (27). A plausible reason for this difference is that the phenotypes we detect depend on the synapses analyzed –we did not study CA1 region output synapses or olfactory granule cell output synapses in the present study– and although the phenotypes observed in the paired recordings in the cortex are very robust (Figures 5-9), we may have, by chance, examined synapses with weaker phenotypes. Finally, it is likely that Cre-mediated recombination is not 100%, leading to an underestimate of the phenotype.

3) Why do morphological and electrophysiological phenotypes sometimes differ in severity? This is observed, for example, for Pv^+^ synapses in the hippocampus (Figure 4). One explanation may be that some synapses may undergo a homeostatic adjustment that renders them stronger. Another explanation could be that the functional analysis in these examples is less specific than the paired recordings performed for Pv^+^ and SST^+^ synapses in the cortex, where the resulting functional and imaging phenotypes are similarly severe (Figures 5-8).

Our study includes inherent limitations that need to be considered in evaluating our conclusions. Frist, we do not know whether the Cre-driver mice and the virally delivered full Cre-mediated recombination of the *Nrxn1, Nrxn2*, and *Nrxn3* genes in our experiments. This question is difficult to address experimentally because the targeted cells in our experiments mostly involve a small subset of neurons. The fact that in a previous study (72), the sextuple KO of PTPRs and neurexins causes a massive phenotype indicates that Pv-Cre-mediated recombination is effective but proving the extent of this recombination is technically challenging.

Second, the *Nrxn1* deletion we analyzed does not abolish expression of *Nrxn1γ*, which does not bind to any of the known neurexin ligands except for FAM19A’s and CA10/11 (6, 73). At least in C. elegans, which expresses a single α- and γ-neurexin but lacks β-neurexin, the γ-neurexin is functionally important (7). Thus, it is possible that some of the functions of neurexins were not detected in our analysis because the expression of *Nrxn1*γ persists.

Finally, it has recently become increasingly clear that paralogs of genes do not necessarily perform comparable functions. The three α- and β-neurexin genes are highly homologous and bind to the same ligands but, when analyzed in the same synapses, appear to have distinct functions (23, 24). The key here is the fact that nearly all neurons co-express all seven principal neurexins (the α-, β-, and γ-neurexins) but in different ratios and splice variants. Deleting individual neurexins causes distinct phenotypes whenever studied in detail. As a result, the double and triple deletions produce not simply the additive or even synergistic effects of single deletions but may also cause unpredictable functional interactions.

### Summary.

Consistent with earlier results, our data indicate that neurexins perform a limited essential role in establishing synapse formation but are essential for shaping synapses in a manner that depends on the identity of the pre- and postsynaptic neuron. In SST^+^ interneuron synapses, neurexins couple Ca^2+^-channels to presynaptic release sites as shown earlier for other synapses (11, 49). In cortical Pv^+^ interneuron synapses, neurexins are required for assembly of functional synapses but not for coupling Ca^2+^-channels to presynaptic release sites. The phenotypes are severe in each type of synapse, suggesting that neurexins are organizers and not modulators of synapses. Moreover, the phenotypes are too different between different types of synapses to be attributed to a similar basic mechanism. A molecular dissection of the function of each neurexin in different types of synapses will likely be required to understand why their functions are different, which will then allow relating the particular molecular mechanism involved to the overall design of synapses. In general, however, it is clear that neurexins do not perform just one unitary function, with the lack of apparent roles for neurexins overall in some synapses possibly due to a mixture of functions that balance each other. Thus, the key conclusion of our study comparing defined synapses in three brain regions is that neurexins as a whole perform distinct functions in different types of synapses and brain regions.

## Materials and Methods

Detailed information on all reagents, antibodies, software, and other resources used in this study is provided in Table S1.

### Experimental model: *Nrxn123* triple cKO mice

The use and care of animals complied with the guidelines of Administrative Panel on Laboratory Animal Care at Stanford University and the Animal Care and Use Committee of Huazhong University of Science and Technology, China. All mouse strains, genetic crosses and primers used for genotyping could be found in the SI Appendix, Extended Methods.

### Viruses and In Vivo Injections.

Lentiviruses and AAVs were produced as described (56, 74, 75). Lentiviruses expressing EF1a-NLS-HA-ΔCre-P2A-tdTomato-T2A-EGFP::Syb2 or EF1a-NLS-HA-Cre-P2A-tdTomato-T2A-EGFP::Syb2 were stereotactically injected into the inferior olive at P1 as described (76). AAVs expressing Ef1**α**-DIO-eYFP (AAV-DJ), Ef1**α**-DIO-mCherry (AAV5) and CAG-DIO-ChR2-tdTomato (AAV-DJ) were stereotactically injected into the mPFC at P21 as described (77). See detailed methods in SI Appendix, Extended Methods.

### Electrophysiology

Slices (300 μm thick) were prepared using a vibratome (LEICA VT1200S) as described previously (78). For climbing-fiber (CF)-EPSC recordings (Figure 3G-M and Figure S1), sagittal slices (300 μm thick) of the cerebellum were prepared from mice at postnatal day 35 (P35). Picrotoxin (50 μM) and CNQX (2 μM) were added to the extracellular solution during whole-cell recordings. For the recordings of inhibitory synaptic transmission in the CA1 (Figure 4C-N), horizontal slices (300 μm thick) of the hippocampus were prepared from mice at approximately eight-weeks old. D-APV (50 μM) and CNQX (20 μM) were added to the extracellular solution during whole-cell recordings. Coronal mPFC slices were cut at P35-40. Paired recordings (Figure 5F-J and 8J-N) were recorded in Pv^+^ or SST^+^ interneurons (in current-clamp mode) and nearby connected pyramidal (Pyr) cells. Miniature IPSCs (mIPSC) was recorded in pyramidal cells in presence of 1 μM TTX, 20 μM CNQX and 50 μM D-APV. See detailed methods in SI Appendix, Extended Methods.

### Two-photon Ca^2+^ imaging

For Figures 6 and 9, we utilized Fluo-5F (200 μM) as a Ca^2+^ indicator to monitor Ca^2+^ changes. Alexa Fluor 594 (50 μM) was added into the internal solution for visualization of the cell morphology. See detailed methods in SI Appendix, Extended Methods.

### Immunohistochemistry

Immunohistochemical experiments were performed as previously described (72). Primary antibodies (guinea pig anti-vGluT1, 1:1000; guinea pig anti-vGluT2, 1:500; mouse anti-Calbindin, 1:500; rabbit anti-Calbindin, 1:500; rabbit anti-GFP, 1:1000; rabbit anti-vGAT, 1:500; rabbit anti-Pv, 1:2000) and secondary antibodies (1:1000, Invitrogen, Alexa 488, Alexa 546, or Alexa 633) were used for immunolabeling. See detailed methods in SI Appendix, Extended Methods.

### Confocal image acquisition and analysis

Images (at 1024 × 1024 resolution) were acquired using a Nikon confocal microscope (A1Rsi) with a 60X oil objective (PlanApo, NA1.4). The general analysis module in NIS-Elements Advanced Research software (Nikon) or ImageJ software was used. See detailed methods in SI Appendix, Extended Methods.

### STED Microscopy

STED imaging was performed as previously described (27). Immunolabeling was performed using guinea pig anti-vGluT2 (1:500) as the primary antibody, followed by incubation with STAR ORANGE-conjugated secondary antibody (1:500). See detailed methods in SI Appendix, Extended Methods.

### Single-Cell Transcriptional Profiling

For Figure 10, Single-Cell Transcriptional Profiling was performed as previously described (45). Primer and probe sequences used for the single-cell analyses are listed in Table S2. See detailed methods in SI Appendix, Extended Methods.

### Quantifications and statistical analyses

All data are means ± SEM; numbers of neurons, sections, or ROIs /mice analyzed are shown in the bars or plots. Inter-group comparisons were performed using two-tailed Student’s *t*-test for comparisons of two conditions (bar diagrams) or Kolmogorov-Smirnov test for cumulative distributions, multiple comparisons were analyzed with two-way ANOVA with Bonferroni’s post-test (*p<0.05, **p<0.01, and ***p<0.001). Image backgrounds were normalized using the same settings for all conditions in an experiment, and immunoreactive elements were analyzed with the Nikon analysis software. Test and controls were analyzed on anonymized samples to prevent observer bias. All experiments were performed with at least three independent biological replications.

## Figures and Tables

**Table 1.** Properties of synapses formed by Pv^+^ or SST^+^ interneurons on pyramidal neurons in layer 5 of the mPFC as revealed by paired recordings.

Properties	Pv^+^ control (n = 26/6)	Pv^+^ *Nrxn123* cKO (n = 46/11)	SST^+^ control (n = 10/5)	SST^+^ *Nrxn123* KO (n = 7/4)
Connection	36.3%	36.8%	25.6%	11.5%
probability	(32/88 tested)	(52/141 pairs)	(11/43 pairs)	(7/61 pairs)
Failure rate	3.0 ± 1.7%	3.2 ±1.7%	30.5 ± 7.6%	27.1 ±8.0%
Latency (ms)	0.96 ± 0.04	1.06 ± 0.06	1.58 ± 0.09	2.23 ± 0.11 (***)
SD of latency (ms)	0.19 ± 0.04	0.28 ± 0.04	0.51 ± 0.05	0.66 ± 0.07
Amplitude (pA)	269.3 ± 50.7	157.6 ± 22.1 (*)	27.7 ± 3.8	17.1 ± 0.8 (*)
CV of amplitude	0.27 ± 0.02	0.34 ± 0.02	0.33 ± 0.02	0.34 ± 0.04
Rise time (ms)	0.84 ± 0.07	0.85 ± 0.06	1.83 ± 0.22	2.80 ± 0.24(*)
Decay time (ms)	18.5± 0.9	18.9 ± 0.8	17.0± 1.7	20.1 ± 2.2

Student’s t-test was performed between control and cKO pairs for PV and SST cells, respectively

* = P<0.05

*** = P<0.001; number of connected pairs/mice analyzed are listed on top.

## Data Availability

All raw data are publicly available at (SDR: https://purl.stanford.edu/xp149zq3345)
